# Therapeutic Trial of anle138b in Mouse Models of Genetic Prion Disease

**DOI:** 10.1128/jvi.01672-22

**Published:** 2023-01-18

**Authors:** Sonia M. Vallabh, Dan Zou, Rose Pitstick, Jill O’Moore, Janet Peters, Derek Silvius, Jasna Kriz, Walker S. Jackson, George A. Carlson, Eric Vallabh Minikel, Deborah E. Cabin

**Affiliations:** a Stanley Center for Psychiatric Research, Broad Institute of MIT and Harvard, Cambridge, Massachusetts, USA; b McCance Center for Brain Health, Massachusetts General Hospital, Boston, Massachusetts, USA; c Department of Neurology, Massachusetts General Hospital, Boston, Massachusetts, USA; d Department of Neurology, Harvard Medical School, Boston, Massachusetts, USA; e Prion Alliance, Cambridge, Massachusetts, USA; f Montana Veterinary Diagnostic Laboratory, Bozeman, Montana, USA; g McLaughlin Research Institute, Great Falls, Montana, USA; h Cervo Brain Research Center, Université Laval, Québec, Québec, Canada; i Department of Biomedical and Clinical Sciences, Linköping University, Linköping, Sweden; j Institute for Neurodegenerative Diseases, University of California—San Francisco, San Francisco, California, USA; Cornell University

**Keywords:** drug development, neurodegeneration, prion

## Abstract

Phenotypic screening has yielded small-molecule inhibitors of prion replication that are effective *in vivo* against certain prion strains but not others. Here, we sought to test the small molecule anle138b in multiple mouse models of prion disease. In mice inoculated with the RML strain of prions, anle138b doubled survival and durably suppressed astrogliosis measured by live-animal bioluminescence imaging. In knock-in mouse models of the D178N and E200K mutations that cause genetic prion disease, however, we were unable to identify a clear, quantifiable disease endpoint against which to measure therapeutic efficacy. Among untreated animals, the mutations did not impact overall survival, and bioluminescence remained low out to >20 months of age. Vacuolization and PrP deposition were observed in some brain regions in a subset of mutant animals but appeared to be unable to carry the weight of a primary endpoint in a therapeutic study. We conclude that not all animal models of prion disease are suited to well-powered therapeutic efficacy studies, and care should be taken in choosing the models that will support drug development programs.

**IMPORTANCE** There is an urgent need to develop drugs for prion disease, a currently untreatable neurodegenerative disease. In this effort, there is a debate over which animal models can best support a drug development program. While the study of prion disease benefits from excellent animal models because prions naturally afflict many different mammals, different models have different capabilities and limitations. Here, we conducted a therapeutic efficacy study of the drug candidate anle138b in mouse models with two of the most common mutations that cause genetic prion disease. In a more typical model where prions are injected directly into the brain, we found anle138b to be effective. In the genetic models, however, the animals never reached a clear, measurable point of disease onset. We conclude that not all prion disease animal models are ideally suited to drug efficacy studies, and well-defined, quantitative disease metrics should be a priority.

## INTRODUCTION

Prion disease is a fatal neurodegenerative disease caused by the conformation conversion of the cellular prion protein, PrP^C^, into a misfolded conformer known as scrapie prion protein or PrP^Sc^ (1). Therapeutics currently in development for prion disease aim to either decrease PrP^C^ expression (2, 3) or bind PrP^C^ (4). Phenotypic screening in prion-infected mouse neuroblastoma cells has also yielded small molecules that inhibit PrP^Sc^ accumulation without targeting PrP^C^ (5). Of these small-molecule leads, anle138b (6) has recently cleared a phase I clinical trial in healthy volunteers (7) and is currently in clinical development for synucleinopathies (ClinicalTrials.gov identifier NCT04685265). Because anle138b was previously shown to be effective against the RML strain of prions in wild-type (WT) mice (6), we sought to determine whether it would be effective in mouse models of genetic prion disease.

The knock-in mouse lines ki-3F4-FFI (8) and ki-3F4-CJD (9) harbor mutations orthologous to D178N and E200K, two of the three most common (10) causes of genetic prion disease in humans. Compared to inoculated models of prion disease, we envisioned that a therapeutic trial in a genetic mouse model would serve two goals. First, we could evaluate efficacy across different prion strains or subtypes, which is important because some antiprion drug candidates have proven to be strain specific (11). Second, we could evaluate efficacy in a spontaneously sick model, which might offer a unique opportunity to modulate prion initiation in addition to prion replication, which is relevant because individuals at risk for genetic prion disease appear to be negative for pathological markers for most of their lives (12, 13). We selected the ki-3F4-FFI and ki-3F4-CJD models because their mutations represent common causes of genetic prion disease in humans (14, 15), and they do not overexpress PrP (8, 9). As a disease endpoint, we chose live-animal bioluminescence imaging wherein a Tg(Gfap-luc) transgene (16) couples luciferase expression to astrocytosis. *Gfap* is among the earliest-upregulated genes in prion-inoculated mice (17, 18), and live-animal imaging provides a real-time readout of this pathology (19); aged ki-3F4-FFI mice were likewise reported to develop strong glial fibrillary acidic protein (GFAP) immunoreactivity in the cerebellum and thalamus (8).

## RESULTS

**FIG 1:**
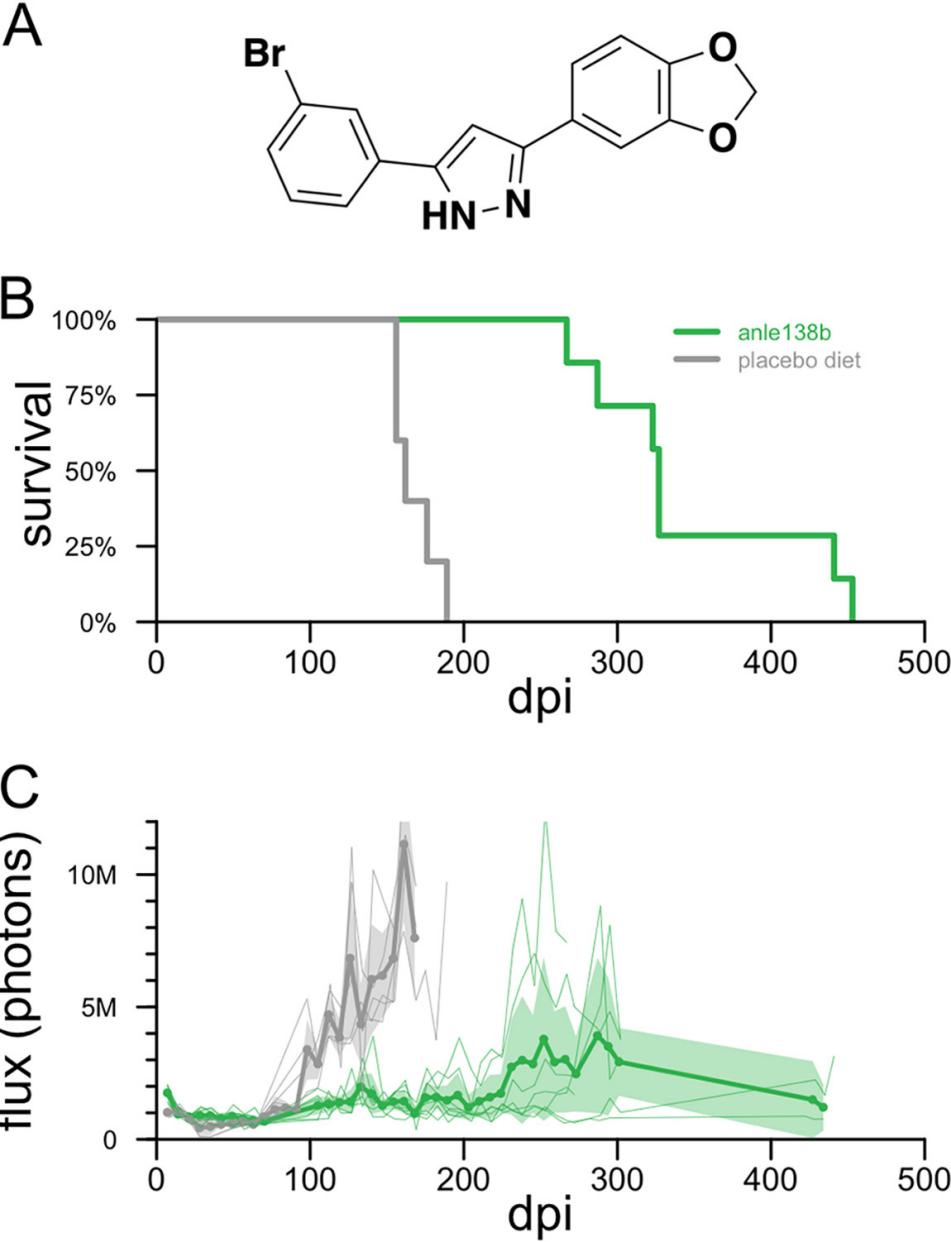
Efficacy of anle138b in RML prion-infected mice. (A) Structure of anle138b. (B) All-cause mortality for 5 placebo diet- and 7 anle138b-treated Tg(Gfap-luc) mice with wild-type *Prnp* genes. (C) Bioluminescence trajectories for the same animals. Thin lines indicate individual animal trajectories, thick lines and dots indicate means per week per treatment group, and shaded regions indicate 95% confidence intervals of the means. Animals were imaged approximately weekly through ~300 dpi; the two long-surviving treated animals were imaged again in two final sessions near the end of life.

As a positive control to ensure the potency of the formulation of anle138b in chow and the utility of our live-animal imaging readout, we first tested the efficacy of anle138b in Tg(Gfap-luc) mice with wild-type *Prnp* genes inoculated with the RML strain of mouse prions. anle138b (Fig. 1A) doubled survival (346 ± 72 versus 168 ± 14 days postinoculation [dpi] [*n* = 7 versus 5] [*P* = 0.00027 by a log rank test]) (Fig. 1B). The treatment also suppressed astrogliosis, delaying and, in some animals, preventing the increase in the bioluminescence signal seen in control animals (Fig. 1C); the peak bioluminescence reached before death was 45% lower in treated animals (*P* = 0.015 by a Kolmogorov-Smirnov test).

**FIG 2:**
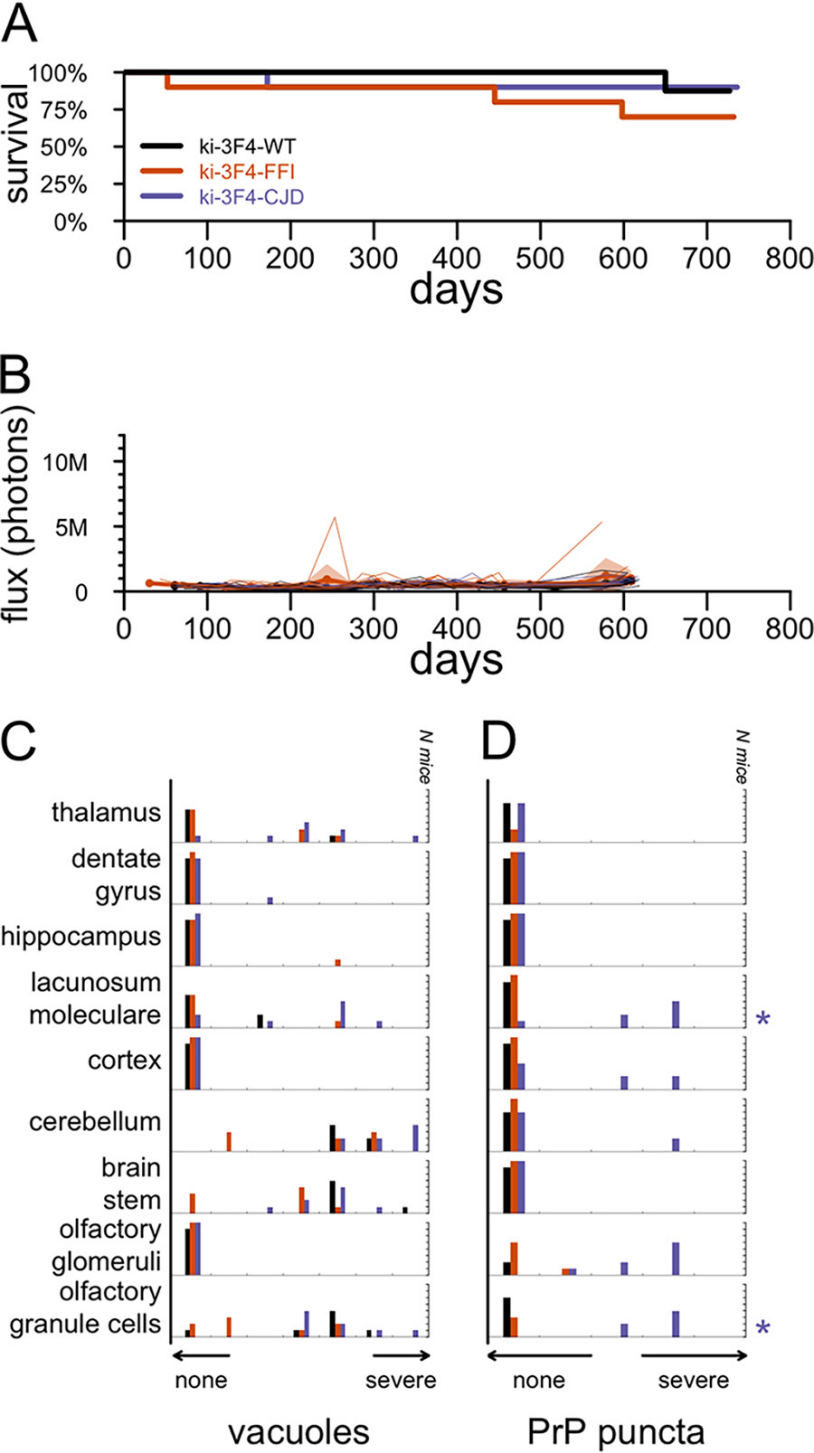
Candidate endpoints in placebo-treated knock-in mice. (A) All-cause mortality for 10 animals per group through 719 days of age. (B) Bioluminescence trajectories for the same animals. Animals were imaged approximately monthly, and occasionally twice monthly, through approximately 600 days of age. Thin lines indicate individual animal trajectories, thick lines and dots indicate means per week per treatment group, and shaded regions indicate 95% confidence intervals of the means. For ease of comparison to RML prion-infected mice, the *y* axis scale is the same as that in Fig. 1B. (C and D) Histogram of vacuolization scores (C) and histogram of 3F4 (PrP) punctum scores (D) for 5 to 8 animals per group subjected to histology analysis. Distributions for ki-3F4-FFI and ki-3F4-CJD mice were compared to those for WT mice for each region using Wilcoxon tests, and multiple testing was corrected using false discovery rates. Symbols at the right indicate corrected statistical significance (*, FDR of <5%; **, FDR of <1%) and are color-coded by genotype.

**FIG 3:**
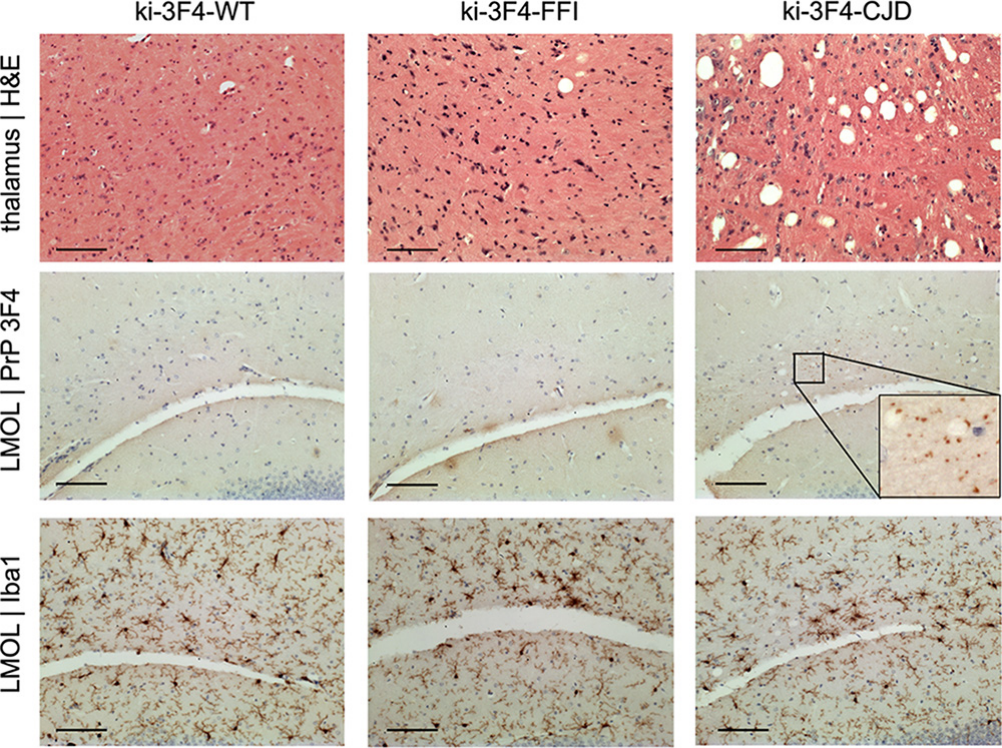
Example histological findings in placebo-treated knock-in mice. Rows show (from top to bottom) vacuolization, 3F4 (PrP) immunoreactive puncta, and Iba1 (microglia); columns show one animal each from the WT, ki-3F4-FFI and ki-3F4-CJD placebo-treated groups. LMOL, lacunosum moleculare layer of the hippocampus; H&E, hematoxylin and eosin. Bars, 100 μm.

Whereas RML-inoculated animals reach a terminal disease endpoint supportive of survival as a primary endpoint, the ki-3F4-FFI and ki-3F4-CJD knock-in models were reported to develop pathology in the absence of terminal illness (8, 9). We therefore prioritized histological and bioluminescence outcomes based on the assumption that most animals would survive to the end of the study. Knock-in animals were monitored out to 665 to 751 days of age before being harvested for histology. Among animals on a placebo diet, the overall survival assessed out to 719 days did not differ among the three genotypes (*P* = 0.43 by a log rank test) (Fig. 2A). In contrast to RML-inoculated mice (Fig. 1C), the bioluminescence for the three knock-in lines on a placebo diet remained low and indistinguishable through 600 days of age (Fig. 2B). At sacrifice, placebo-treated animals were scored for vacuolization and PrP punctum (3F4) staining across 9 brain regions (Fig. 2C and D). After correction for multiple testing, ki-3F4-CJD animals exhibited 3F4 punctum scores that were significantly elevated above those of the ki-3F4-WT controls in the lacunosum moleculare layer of the hippocampus as well as in olfactory granule cells (false discovery rate [FDR] of <5% by a Wilcoxon test). No other differences were statistically significant at the group level, although light vacuolization could be observed in some ki-3F4-FFI and ki-3F4-CJD animals, particularly in the thalamus (Fig. 3). 3F4 staining was overall faint in ki-3F4-FFI animals (Fig. 3), consistent with the underexpression of PrP and the lack of clear deposition in this line (8). Microglial activation assessed through Iba1 staining in select animals generally appeared to be at normal levels for aged animals and was not different between the knock-in lines (Fig. 3). In a combined model accounting for individual animal and regional differences (see Materials and Methods), vacuolization was significantly enriched in ki-3F4-CJD animals (*P* = 0.012 for ki-3F4-CJD, and *P* = 0.060 for ki-3F4-FFI, each versus ki-3F4-WT). Puncta likewise appeared enriched in ki-3F4-CJD animals (the *P* value was not defined because no puncta were observed in any ki-3F4-WT mice).

**FIG 4:**
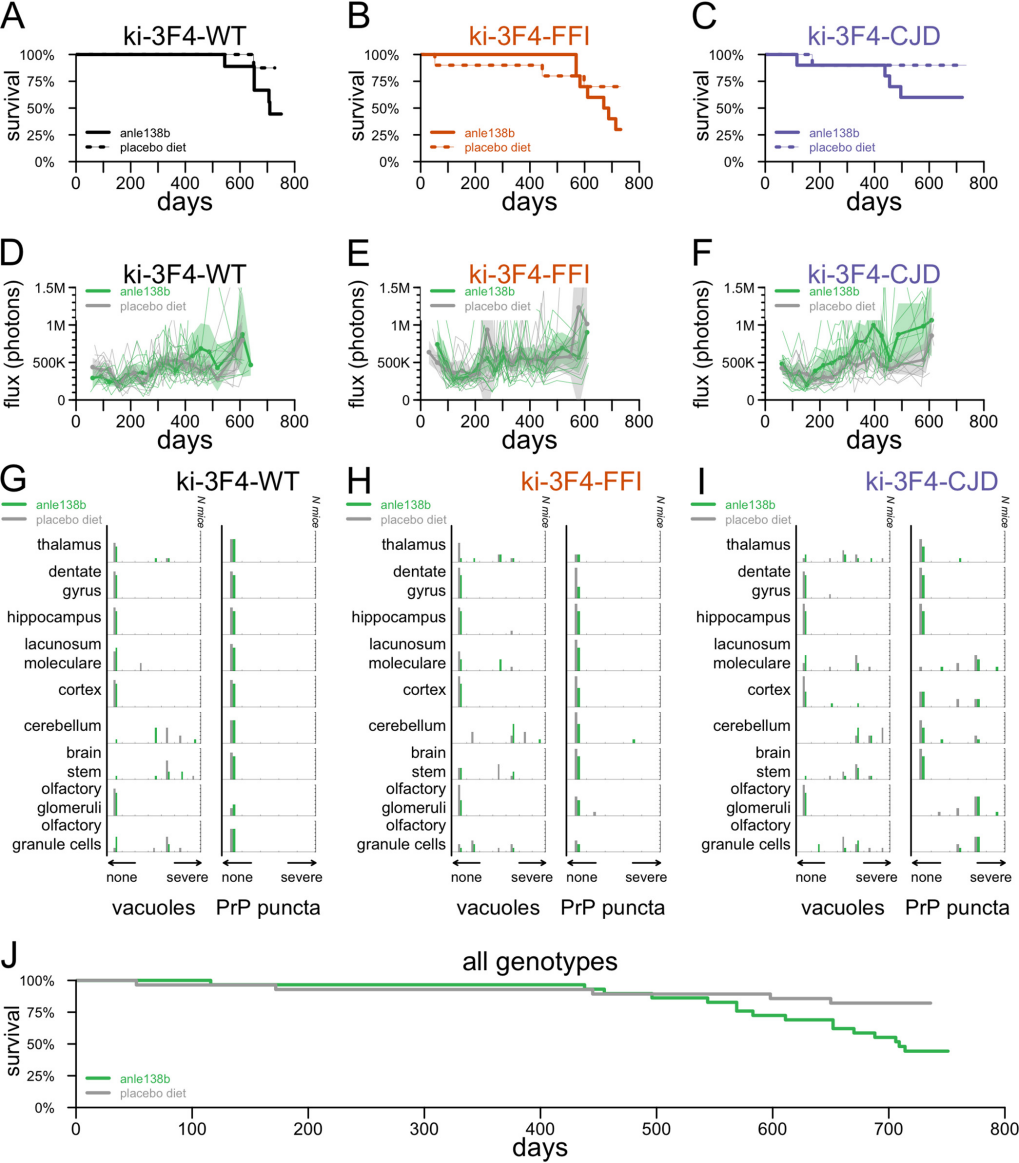
Effect of anle138b on endpoints in knock-in mice. (A to I) All-cause mortality (A to C), bioluminescence trajectories (D to F), and histology endpoints (G to I) in anle138b- versus placebo-treated animals of genotypes ki-3F4-WT (A, D, and G), ki-3F4-FFI (B, C, and H), and ki-3F4-CJD (C, F, and I). (J) Combined all-cause mortality curve ignoring genotype.

**FIG 5:**
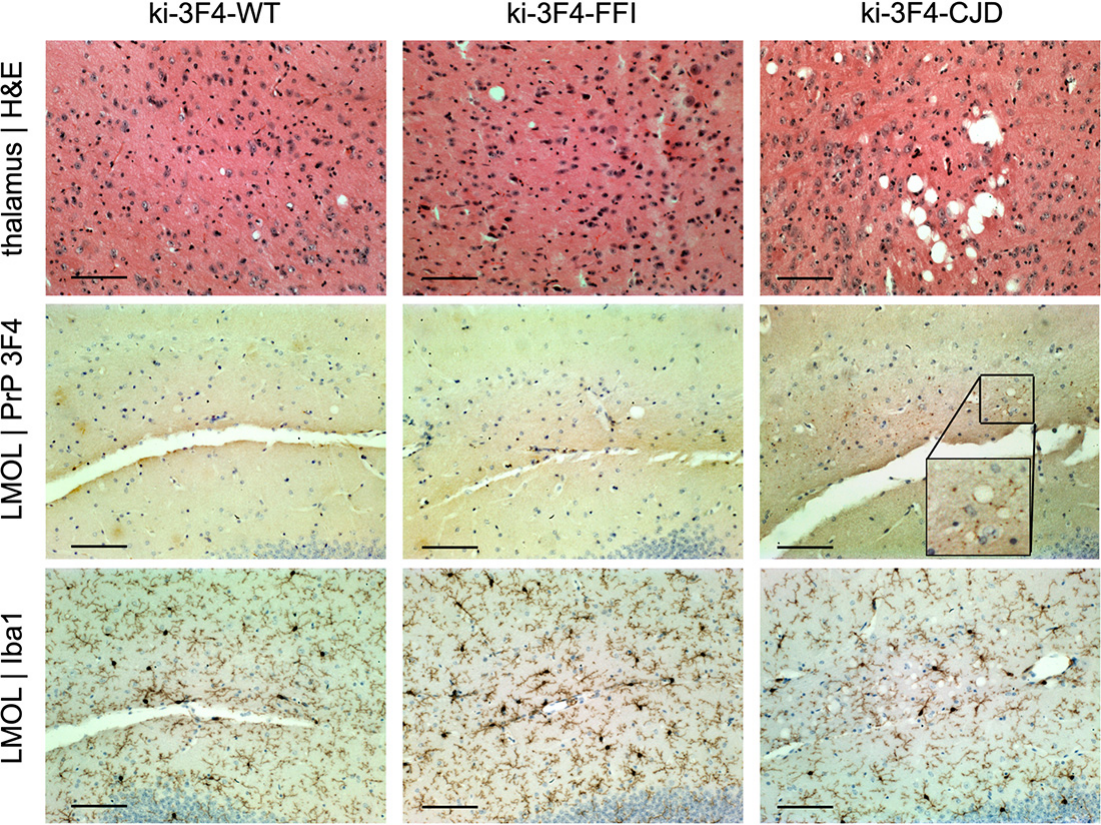
Histology in anle138b-treated knock-in mice. As in Fig. 3, rows show (from top to bottom) vacuolization, 3F4 (PrP) immunoreactive puncta, and Iba1 (microglia); columns show one animal each from the WT, ki-3F4-FFI and ki-3F4-CJD anle138b-treated groups. LMOL, lacunosum moleculare layer of the hippocampus. Bars, 100 μm.

The overall survival of knock-in animals treated with anle138b was not distinguishable from that of matched controls within any one genotype (*P* = 0.093 for ki-3F4-WT [Fig. 4A], *P* = 0.23 for ki-3F4-FFI [Fig. 4B], and *P* = 0.15 for ki-3F4-CJD [Fig. 4C]). In a combined analysis, anle138b-treated animals appeared to exhibit poorer survival than control animals (*P* = 0.11 with genotype as a covariate, or *P* = 0.0087 ignoring genotype, by log rank tests) (Fig. 4J). Bioluminescence trajectories were similarly low for all groups regardless of genotype and treatment group (Fig. 4D to F). Histological analysis yielded no significant differences in vacuolization or PrP puncta when comparing treatment groups within genotypes (Fig. 4G to I), and in a combined model (see Materials and Methods), neither outcome differed significantly by treatment group (*P* = 0.50 for vacuoles, and *P* = 0.25 for puncta). Neuropathology in anle138b-treated mice (Fig. 5) looked qualitatively similar to that in placebo-treated animals.

## DISCUSSION

Our pilot study in mice infected with the RML strain of prions replicated the survival benefit of anle138b treatment in this model (6) and further demonstrated the treatment’s impact on a bioluminescence readout serving as a proxy for reactive astrogliosis. Interestingly, the response of prion disease-associated astrogliosis to therapy may depend on the drug’s mechanism of action. In animals treated with a single dose of PrP-lowering antisense oligonucleotide, astrogliosis remained low even as the drug washed out and animals eventually developed terminal prion disease (3), and GFAP staining was reduced in treated animals that reached the endpoint (2). In contrast, the small-molecule PrP^Sc^ accumulation inhibitor compound B delayed the onset of astrogliosis, but treated animals ultimately reached bioluminescence levels equal to or even higher than those of untreated controls (20). In the present study of anle138b, astrogliosis in treated animals remained low on average, even as treated animals eventually succumbed to prion disease. This difference in averages is due in part, however, to the more variable time of onset among treated animals; some but not all treated animals did display a clear increase in bioluminescence before death. It will be interesting to determine whether and how the distinct mechanisms of action of antiprion therapies might lead to differences in end-stage pathology.

PrP-lowering therapy appears to be effective across prion strains (3), whereas small molecules identified through phenotypic screening have so far proven effective against some prion strains but not others (11, 20–23) and can engender drug resistance (11, 24). Despite its efficacy in RML prion-infected mice, anle138b proved ineffective in humanized mice infected with sporadic Creutzfeldt-Jakob disease type MM1 prions (22). In a mouse model of the A117V mutation, anle138b was reported to reduce the plaque load without improving survival (25). Here, we sought to test the efficacy of anle138b in two knock-in mouse models of genetic prion disease corresponding to the E200K and D178N mutations. Unexpectedly, we were unable to rigorously evaluate the efficacy of anle138b because none of the selected endpoints provided a clear, quantitative measure of disease in these mice. To the extent that we were able to evaluate anle138b, we found no evidence that it modified neuropathology in these mouse models, and if anything, anle138b-treated animals survived for a shorter period than placebo controls. These knock-in models do not reach a terminal prion disease endpoint, so this survival difference, if real, would not likely reflect a worsening of prion disease. Rather, it may simply point to long-term toxicity at the high dose used here, 2 g per kg of chow formulated in chow. For a 32-g animal consuming 4 g of feed per day, this corresponds to a dose of 250 mg/kg/day. A 28-day toxicology study in cynomolgus macaques established a no-adverse-event limit (NOAEL) of 125 mg/kg/day (7). These mice were exposed to twice that dose for 20 times as long. A phase I clinical trial of anle138b tested doses of just 50 to 300 mg/day in individuals averaging ~80 kg (7), corresponding to 0.6 to 3.75 mg/kg/day, less than 1.5% of the dose used in these mice.

These knock-in mouse lines were previously reported to exhibit differences in body temperature regulation, burrowing, rotarod performance, and certain behavioral phenotypes extracted from video feeds by automated mouse behavioral analysis (AMBA), but a frank difference in all-cause mortality was not reported (8, 9), consistent with our findings here. A consistent increase in GFAP staining by 12 to 16 months was reported for these models (8, 9), inspiring our use of live-animal imaging in Tg(Gfap-luc) mice as an endpoint here. However, subsequent studies did not identify a clear increase in *Gfap* mRNA levels in the whole-brain homogenates from these animals (W. S. Jackson, unpublished observations), mirroring our results. Specific changes in mRNA translation have been observed in these knock-in lines (26), distinct from those observed in RML-inoculated mice (27), but these findings are more recent and were not leveraged here. Various histopathological abnormalities have also been identified in these knock-in mouse lines by 20 months (~600 days) (8, 9), some of which we replicate here. Translating these histological changes into disease endpoints, however, is dependent on staining and imaging conditions and subjective scoring. It can be challenging to consistently translate these into quantitative endpoints. In addition, we cannot rule out that the penetrance or onset of pathological features in the knock-in mice in this study may have been modified by their mixed genetic background, the presence of the *Gfap-luc* allele, or myriad differences in facility or husbandry practices.

It has been proposed that antiprion therapeutics might enter clinical development in presymptomatic individuals at risk for genetic prion disease (28). In this context, efficacy studies in mouse models that develop spontaneous prion disease (29) may seem especially appealing. The present study suggests that such models may in some cases yield invaluable insights into the basic science of prion disease without meeting additional requirements specific to the context of therapeutic studies. In particular, efficacy studies are served by disease endpoints that are quantitative, objective, easily measured, monitorable in real time, highly penetrant, and tightly distributed in severity or onset. Myriad mouse models of spontaneous prion disease are available, often harboring mutations orthologous to those causing genetic prion disease in humans, and these models are variably reported to exhibit certain pathognomonic features of human prion disease, including spongiform change, PrP^Sc^ deposition, transmissibility, and/or fatality (30–51). The potential utility of these models in therapeutic efficacy studies remains to be evaluated, but we can anticipate certain relevant considerations here. Even where disease in these models exhibits full penetrance, the standard deviation (SD) may be on the order of 2 months (50) rather than 1 week as in RML-infected mice, making it more challenging to power studies. Often, the reported endpoint is one of central nervous system (CNS) dysfunction observed through behavioral assays rather than a strictly objective endpoint such as weight loss. This may increase the risk of introducing new variability when transferring these endpoints to new laboratories or personnel. Transgene integration sites in many of these models remain uncharacterized, and caution may be merited regarding potential artifacts (52). Most of these models are overexpressers, at least at the level of the transgene copy number or mRNA expression level. Some mutant PrPs are constitutively underexpressed, so even modest expression levels (50) may actually exceed those observed in human mutation carriers (53). The nonlinear relationship between the PrP dosage and the prion disease incubation period (54) may pose one potential limitation to the use of PrP-overexpressing lines in efficacy studies of PrP-lowering therapeutics in particular.

Given the difficulty in modeling most neurological diseases in animals, the diversity of models and even species available for prion research by virtue of the panmammalian nature of the disease is an unusual endowment. Although this panoply is united by the core prion disease process, the present study may serve as a cautionary reminder that, as we have argued elsewhere with regard to nonhuman primates (55), not all prion models are ideally suited to all research purposes. Ultimately, multiple models will be needed to stitch together the complex continuum that bridges fundamental disease biology to therapeutic hypotheses and, ultimately, drug development.

## MATERIALS AND METHODS

### Study design.

All studies were conducted at the McLaughlin Research Institute between June 2014 and June 2016 under IACUC approval MRI-GAC11. In addition to the mouse orthologs of the D178N and E200K mutations, the ki-3F4-FFI and ki-3F4-CJD alleles also contain the 3F4 epitope common to many mammals other than mice, which allows detection with the 3F4 antibody. To control for any impact that this might have on disease outcomes, we used ki-3F4-WT mice, which also have this epitope but no mutation and do not develop spontaneous disease (8), as a control group. Under the expectation that the onset of astrocytosis would occur at the age of 13 ± 3 months (mean ± SD), a sample size of 10 per group was calculated to provide 80% power (by a 2-sided *t* test) to detect a 30% delay in onset; anle138b in RML prion-infected mice had been reported to delay disease by 30 to 97% depending on the time point of treatment initiation (6). Because mice would be subject to humane euthanasia, we also included survival as an endpoint. Finally, we included histopathology because spongiform changes and PrP deposition are pathognomonic for prion disease. Animal care and histology scoring were performed in an unblind manner.

### Animals.

Prion inoculations were performed by utilizing homozygous Tg(Gfap-luc) mice on an FVB/N background. Experimental knock-in mice were hemizygous Tg(Gfap-luc) and homozygous knock-in mice, resulting in a mixed background of FVB/N (from the Gfap-luc mice) as well as 129/Ola and C57BL/6 (on which the knock-in alleles were created). All cohorts were of mixed sexes.

### Procedures and monitoring.

For the RML inoculation experiment, animals were inoculated intracerebrally with 10 μL of 1% brain homogenate from animals terminally sick with the RML strain (56) of prions using a 26-gauge Hamilton syringe. Knock-in animals were not inoculated. Live-animal imaging was performed in a manner similar to the one described previously (3). Animals were injected intraperitoneally with 150 mg/kg d-luciferin (GoldBio) in a 15-mg/mL solution and imaged in a Lumina II *in vivo* imaging system (IVIS) (PerkinElmer) at 10 to 20 min postinjection. Animals underwent daily monitoring plus welfare checks weekly, escalating to three times weekly at 90 days postinoculation (dpi) for inoculated animals, with euthanasia upon ≥20% weight loss from a 4-month baseline, an inability to reach food or water, difficulty breathing, or dermatitis or fight wounds refractory to treatment. All animals, regardless of the cause of death, were included in survival curves. Due to the retrospective nature of the analysis, there were a total of 6 animals for which records were incomplete: 2 (1 in the knock-in experiment and 1 in the RML inoculation experiment) for which the exact date of death was not recorded and instead was approximated based on the last known observation of the animal and an additional 4 (all in the knock-in experiment) for which records provided the date of death but did not specify the cause or circumstances of death.

### Genotyping.

Gfap-luc mice were genotyped using primer 9020 (TCTCTAAGGAAGTCGGGGAAGC) and primer 9021 (CAGCGGGAGCCACCTGATAGCCTT), with running on 1% agarose with ethidium bromide for an expected product of 430 bp in the presence of the transgene. Knock-in mice were genotyped using primer 75 (GAGCAGATGTGCGTCACCCAG), primer 77 (GAGCTACAGGTGGATAACCCC), and primer 105 (CAACATGAAGCATATGGCA). PCR PP1 using primers 75 and 77 gives a 204-bp product in wild-type mice and a 218-bp product in all three knock-in lines, resolvable with 3 to 4% Tris-acetate-EDTA (TAE) agarose, while heterozygous knock-ins yield a larger heteroduplex. PP38 PCR using primers 105 and 77 yields a product that digests with BbsI for ki-3F4-CJD, yielding bands of 244 and 276 bp; digests with MfeI for ki-3F4-FFI, yielding bands of 210 and 310 bp; and digests with neither enzyme for ki-3F4-WT.

### Compound formulation.

anle138b (CAS no. 882697-00-9) (Fig. 1A) was provided by Armin Giese and was formulated at 2 g/kg in mouse chow by SSNIFF (Soest, Germany). Knock-in animals were weaned onto facility chow and then switched to anle138b or matched placebo diets (SSNIFF) at ~2 months of age (range, 30 to 86 days). In the RML inoculation experiment, controls received facility chow, while treated mice switched to an anle138b diet immediately upon inoculation at 0 dpi.

### Histology.

Antigen retrieval was performed by boiling in 10 mM sodium citrate (pH 6.0) for 10 min. Antibodies were as follows: primary rabbit monoclonal antibody (catalog no. ab178846; Abcam) at 1:2,000 for 3.5 min with diaminobenzidine (DAB) and secondary biotinylated goat anti-rabbit IgG (catalog no. BA1000; Vector Labs) at 5:1,000 for Iba1 and primary mouse monoclonal antibody (catalog no. MAB1562; Millipore) at a 1:1,000 dilution for 5 min with DAB and secondary biotinylated goat anti-mouse IgG (catalog no. BA9200; Vector Labs) at 5:1,000 for PrP 3F4. We utilized a VectaStain Elite ABC-horseradish peroxidase (HRP) kit (catalog no. PK-6200; Vector Labs), Permount mounting reagent (catalog no. SP15; Fisher Scientific), Mayer’s hematoxylin (catalog no. S1275; Sigma), and eosin Y (catalog no. HT110280; Sigma).

### Statistics.

All statistical analyses were conducted using custom scripts in R 4.2.0. All statistical tests were two sided. Survival curves were compared using log rank tests. Bioluminescence trajectories were compared by visualization of 95% confidence intervals; peak bioluminescence values were compared using Kolmogorov-Smirnov tests, which do not assume normality. Histology scores, because they are ordinal variables, were compared primarily using Wilcoxon tests, which do not assume normality and are applicable to rank data. Within each group of regions and endpoints being compared, false discovery rate (FDR) correction was used to control for the multiple-testing burden; an FDR of <5% was considered significant. Because histology scores are ordinal data not well modeled by nested analysis of variance (ANOVA), the overall differences in histology scores across regions were additionally assessed using a cumulative link mixed model using the clmm2 function from the ordinal package in R (57), with score (ordinal) modeled as a function of region, genotype, and, where applicable, treatment group (fixed effects) as well as individual animal (random effects).

### Data availability.

Source code and individual animal-level raw data sufficient to reproduce the figures and analyses here are provided in the publicly available GitHub repository at https://github.com/ericminikel/anle138b.
